# A Frameshift RBM10 Variant Associated With TARP Syndrome

**DOI:** 10.3389/fgene.2022.922048

**Published:** 2022-08-04

**Authors:** Han Daicheng, Xia Shiwen, Zhang Jingxuan, Hu Junbo, Wang Bo

**Affiliations:** ^1^ Department of Neonatology, Maternal and Child Health Hospital of Hubei Province, Wuhan, China; ^2^ Hubei Key Laboratory of Embryonic Stem Cell Research School of Basic Medical Sciences, Hubei University of Medicine, Shiyan, China; ^3^ Department of Pathology, Maternal and Child Health Hospital of Hubei Province, Wuhan, China; ^4^ Department of Clinical Laboratory, Maternal and Child Health Hospital of Hubei Province, Wuhan, China

**Keywords:** TARP, RBM10, X-linked, pulmonary arteriovenous malformation, coagulopathy

## Abstract

TARP syndrome is a rare X-linked genetic condition caused by mutations in the *RBM10* gene. Primary clinical characteristics of TARP syndrome include Talipes equinovarus, Atrial septal defect, Robin sequence and Persistent left superior vena cava. Newly reported cases identified a few novel *RBM10* variants and atypical manifestations associated with TARP syndrome, thus expanding the genetic and clinical spectrum of TARP syndrome. Here we report a molecularly confirmed TARP syndrome with distinctive clinical features including pulmonary arteriovenous malformation, single umbilical artery, and coagulopathy. We identified a frameshift *RBM10* variant that might be associated with his distinctive clinical features.

## Introduction

TARP syndrome is a rare X-linked genetic disorder that causes several birth defects (Niceta et al., 2019). This syndrome was first described in 1970 as Robin’s syndrome (Gorlin et al., 1970). TARP stands for Talipes equinovarus, Atrial septal defect, Persistence of the left superior vena cava and Robin sequence (a set of abnormalities including small lower jaw (micrognathia), displacement of the tongue toward the back of the oral cavity (glossoptosis), dyspnea and an abnormal opening in the roof of the mouth (cleft palate)) (Gorlin et al., 1970). These are the most common clinical features in the early reported cases (Kurpinski et al., 2003).

However, as more patients have been identified by genetic testing, the original TARP acronym doesn’t describe the full phenotypic spectrum of this syndrome. In some cases, patients have only one or two of these features (Powis et al., 2017). Meanwhile, additional clinical features including central nervous system dysfunction, renal abnormalities, cardiac lesions, and distal limb defects have been reported, which demonstrates broad phenotypic heterogeneity among patients with TARP syndrome (Johnston et al., 2010).

TARP syndrome is caused by loss-of-function mutations in the *RBM10* gene (Johnston et al., 2014). This gene maps to chromosome Xp11.23-q13.3 in human, which encodes an RNA binding motif protein that is involved in alternative splicing (Coleman et al., 1996; Thiselton et al., 2002). The function of *RBM10* is not fully understood. *RBM10* has been shown to regulate alternative splicing of pre-mRNA of *NUMB*, *FAS*, *Dlg4*, *SMN2* and Even its own pre-mRNA resulting in alternative splicing-coupled nonsense-mediated mRNA decay (AS-NMD) (Loiselle and Sutherland, 2018). Disease mutations include missense and frameshift mutations that lead to abnormal or truncated proteins (Højland et al., 2018; Imagawa et al., 2020). The identification of *RMB10* variants could help to clarify its clinical variabilities and shed light on the pathogenesis of TARP syndrome.

Here we report a molecularly confirmed TARPS with distinctive clinical features including pulmonary arteriovenous malformation, single umbilical artery, and coagulopathy. We identified a frameshift *RBM10* variant in this patient that might be associated with his distinctive clinical features.

## Methods

### Patients and Samples

A male newborn was delivered by cesarean section because of oligoamnios at a gestational age of 35 weeks and 5 days with a birth weight of 1535 g. The karyotype of his mother is 47, XXX. The parents are Chinese and Han nationality, they had no family history of genetic disease. The mother and the father were 35 and 42 years old respectively at conception. This pregnancy was achieved by *in vitro* fertilization (IVF) due to primary infertility. During the pregnancy, the fetus was diagnosed with small mandible, single umbilical artery and permanent left superior vena cava by ultrasonic testing in 25th week of pregnancy, the mother was diagnosed with oligoamnios and asthma in her 35th week of pregnancy and treated accordingly. Immediately after birth, the newborn was admitted to the neonatal intensive care unit (NICU) because of severe respiratory distress and asphyxia. The baby looked pale and abnormally blue (cyanosis), an indication of poor circulation or insufficient oxygenation of the blood. Because it was difficult to perform endotracheal intubation in the newborn, we gave him maximal ventilator support, with a FiO_2_ of 100%. However, due to the severe coagulopathy and pulmonary hemorrhage, 8 h after birth, the patient died.

### Molecular Analysis

We performed whole-exome sequencing (WES) on the family. The Novaseq6000 platform (Illumina, San Diego, United States), with 150 bp pair-end sequencing mode, was used for sequencing the genomic DNA of the family. The sequencing reads were aligned to the human reference genome (hg38/GRCh38) using the Burrows-Wheeler Aligner tool.

### Autopsy Examination

Autopsy examination were performed including gross anatomy, histologic examinations of the lung biopsy specimen, hematoxylin and eosin (HE) staining, cranial magnetic resonance imaging (MRI) and so on.

## Results

**FIGURE 1:**
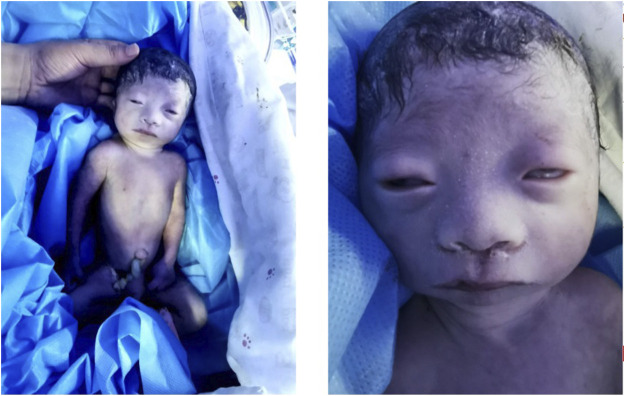
Clinical features. The baby had hypotonia, widely spaced eyes, sparse eyelashes, small lower jaw, wide nasal bridge, wide mouth with downturned corners and low-set ears.

**FIGURE 2:**
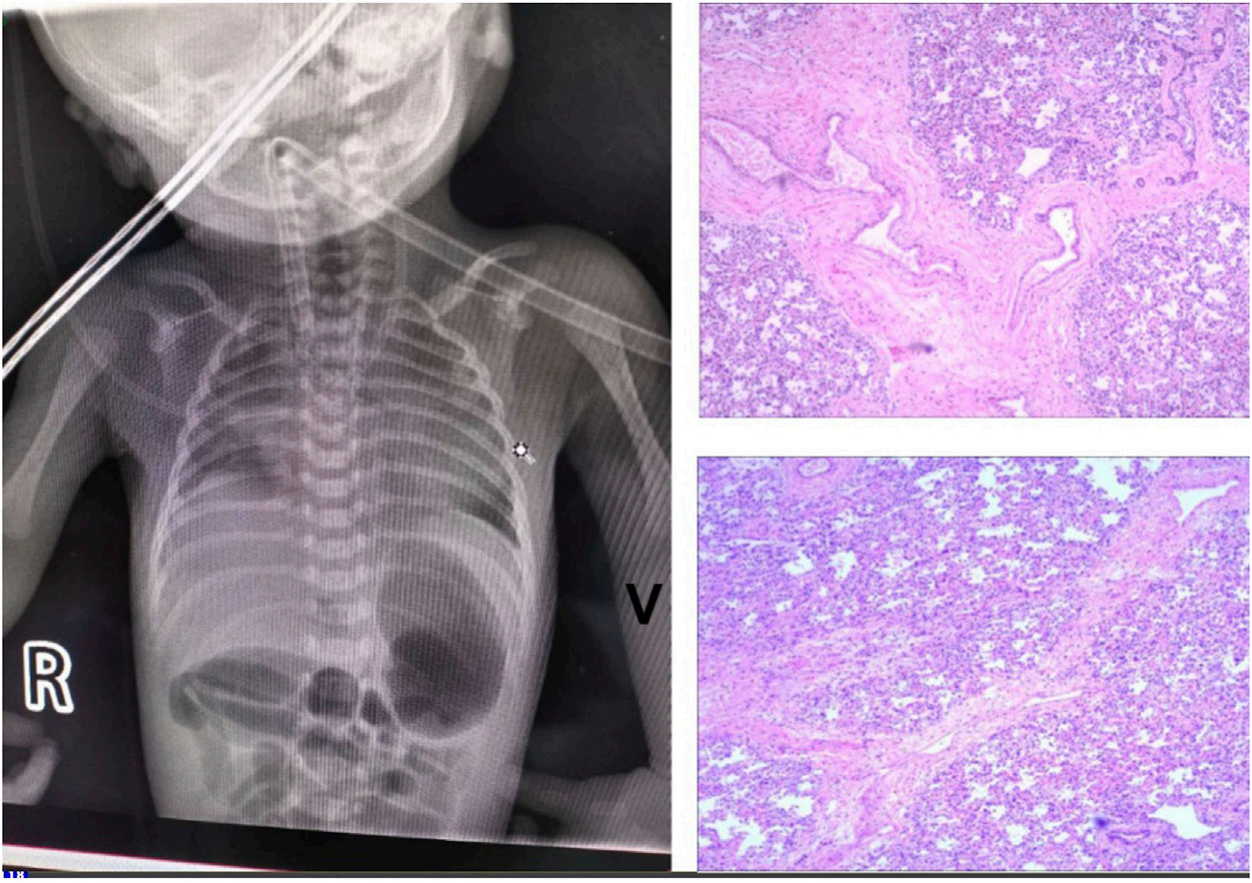
Lung imaging. **(A)** Chest X-Ray showed massive pulmonary pleural effusions and hemorrhage. **(B)** HE staining showed diffusive interstitial fibrosis (*). magnification of ×100. **(C)** Histologic examinations of the lung biopsy specimen demonstrated pulmonary arteriovenous malformations. There was increased number of blood vessels, some developing sac-like dilation and thickening walls (arrows).

This baby had hypotonia and a set of abnormalities including a small lower jaw (micrognathia), displacement of the tongue toward the back of the oral cavity (glossoptosis), and an abnormal opening in the roof of the mouth (cleft palate), which is known as Pierre Robin sequence suggesting a diagnosis of TARP syndrome. He had low-set ears which is consistent with previously reported TARP cases (Figure 1). Further identification of the mutation in the *RBM10* gene established the diagnosis. The patient presented with massive pulmonary pleural effusions and pulmonary hemorrhage (Figure 2) and coagulation disorder (Table 1).

**TABLE 1:** Coagulation tests.

Test	Patient’s result	Normal value
TT (s)	23.3	10.3–16.8
PT (s)	35.5	9.4–12.5
APTT (s)	N/A	25.1–36.5
FIB (g/L)	0.54	2.38–4.98
INR	3.17	0.9–1.2
D-dimer	45.79	<0.5

TT, Thrombin time; PT, prolonged prothrombin time; APTT, activated partial thromboplastin time; FIB, fibrinogen; INR, international normalization ratio.

**FIGURE 3:**
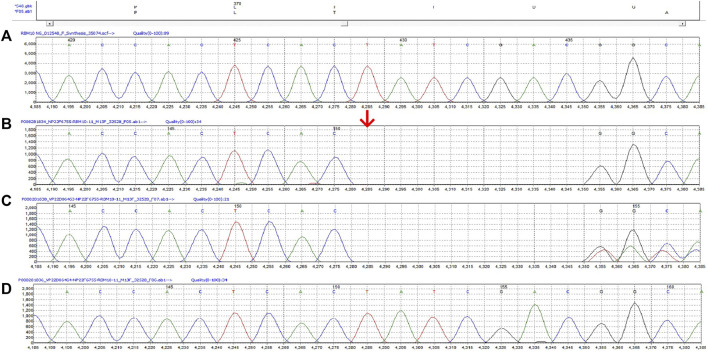
The c.1113_1119del variant in RBM10 of this family. **(A)** The reference sequence **(B)** Proband (hemizygote) **(C)** Mother (heterozygote) **(D)** Father.

The diagnostic whole-exome sequencing showed the patient was hemizygous for a c.1113_1119del variant in *RBM10*. This mutation creates a frameshift at Ile372. We detected the same mutation in the mother and the parents had no family history of genetic disease (Figure 3).

Autopsy examination were performed 48 h after death and showed persistent left superior vena cava and pulmonary arteriovenous malformation, which were responsible for his death. The autopsy also revealed massive bilateral pleural effusions. Histologic examinations of the lung biopsy specimen demonstrated pulmonary arteriovenous malformations. There was increased number of blood vessels, some developing sac-like dilation with thickening walls. HE staining showed diffusive interstitial fibrosis (Figure 2).

**FIGURE 4:**
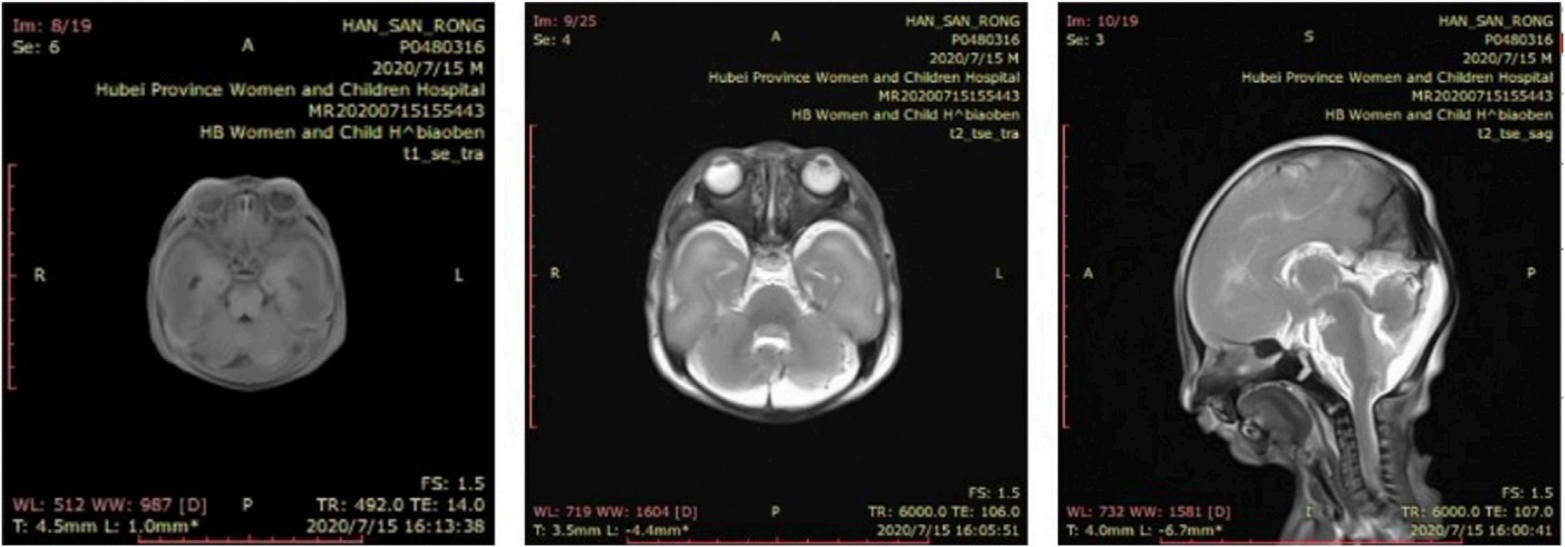
Brain imaging. Cranial MRI showed small lower jaw, displacement of the tough toward the back of the oral cavity and cleft palate and lissencephaly. There was no gyri/sulci clearly seen for both cerebral and cerebellum cortex. **(A)** T1-weighted coronal image **(B)** T2-weighted coronal image **(C)** T2-weighted sagittal image.

Cranial MRI during autopsy detected small lower jaw, displacement of the tough toward the back of the oral cavity and cleft palate and lissencephaly (Figure 4). There was no gyri/sulci clearly seen for both cerebral and cerebellum cortex which are structural brain abnormalities frequently reported in TARP patients (Figure 4).

## Discussion

TARP syndrome is a rare development defect during embryogenesis. Most affected males have died before or shortly after birth, usually due to various heart conditions associated with the disease (Kurpinski et al., 2003). There have been a few exceptional cases of long-term survival (Gripp et al., 2011). In our case, the patient died 8 h after birth, probably due to severe coagulopathy and massive pulmonary hemorrhage. The classic features of TARP syndrome described in the early reported cases include club foot, atrial septal defect, Robin sequence, and persistent left superior vena cava. However, newly reported cases demonstrate significant variability in clinical manifestations (Kaeppler et al., 2018; Imagawa et al., 2020). In some cases, cortical visual impairment, profound intellectual disability, and chronic lung disease are observed. Our patient showed recognizable Robin sequence and persistent left superior vena cava but lacks other features of TARP syndrome. Brain abnormalities including cerebellar hypoplasia and mega cisterna magna, which are frequent in TARP syndrome (Johnston et al., 2014) were absent in our patient. Instead, our patient had pulmonary arteriovenous malformation, single umbilical artery, and coagulopathy, which have not been reported before.

*RBM10* mutations are the cause of TARP syndrome (Johnston et al., 2014). Phenotypic diversity of this condition is thought to be the result of genetic variations of the *RBM10* gene including frameshift, nonsense, and deletion mutations (Wang et al., 2013). We identified a frameshift mutation (c.1113_1119del, p. Ile372fs) in exon 11 of the *RBM10* gene. Although frameshift mutations are in general cause the reading of the codons after the mutation to code for different amino acid resulting in loss-of-function protein, different frameshift mutants contribute to different phenotypes in patients with TAPR syndrome. Unlike other studies showing various heart conditions and brain abnormalities associated with TARP syndrome, we found this *RBM10* variant was associated with severe coagulopathy and pulmonary arteriovenous malformation.

Previous functional analysis of six *RBM10* mutations that predicted to be pathogenic from the COSMIC database including one nonsense mutation, four missense mutation, and one frameshift mutation F227fs*39 (c.678delC)) found that when expressed in HEK293, the expressions of nonsense and frameshift mutation were absent demonstrating they lead to non-sense-mediated decay (Zhao et al., 2017). Another study using mouse cells has shown that *RBM10* deletion could lead to splicing changes of multiple target genes that affect normal palate development and cause human disease (Rodor et al., 2017). To clarify the role of this mutant in the pathogenesis of the disease, it would be interesting the perform the functional analysis of this mutant *in vitro* or in animal models of lung injury or alveolar hemorrhage.

To conclude, we identified a *RBM10* variant associated with TARP syndrome. Awareness of the expanded phenotypic spectrum will improve the diagnosis and genetic counselling of TARP syndrome.

## Data Availability

The datasets for this article are not publicly available due to concerns regarding participant/patient anonymity. Requests to access the datasets should be directed to the corresponding author.
